# The *Aspergillus nidulans* Kinesin-3 Tail Is Necessary and Sufficient to Recognize Modified Microtubules

**DOI:** 10.1371/journal.pone.0030976

**Published:** 2012-02-20

**Authors:** Constanze Seidel, Nadine Zekert, Reinhard Fischer

**Affiliations:** Department of Microbiology, Institute for Applied Biosciences, Karlsruhe Institute of Technology, Karlsruhe, Germany; Cinvestav, Mexico

## Abstract

Posttranslational microtubule modifications (PTMs) are numerous; however, the biochemical and cell biological roles of those modifications remain mostly an enigma. The *Aspergillus nidulans* kinesin-3 UncA uses preferably modified microtubules (MTs) as tracks for vesicle transportation. Here, we show that a positively charged region in the tail of UncA (amino acids 1316 to 1402) is necessary for the recognition of modified MTs. Chimeric proteins composed of the kinesin-1 motor domain and the UncA tail displayed the same specificity as UncA, suggesting that the UncA tail is sufficient to establish specificity. Interaction between the UncA tail and alpha-tubulin was shown using a yeast two-hybrid assay and in *A. nidulans* by bimolecular fluorescence complementation. This is the first demonstration of how a kinesin-3 motor protein distinguishes among different MT populations in fungal cells, and how specificity determination depends on the tail rather than the motor domain, as has been demonstrated for kinesin 1 in neuronal cells.

## Introduction

The microtubule (MT) cytoskeleton is assembled from alpha, beta-tubulin heterodimers. In addition, multiple isoforms and posttranslationally modified tubulins (PTMs) are known [1]. For instance, certain neuronal cells use alpha-tubulin where the C-terminal tyrosin is cleaved (detyrosinated alpha-tubulin) [2]. Other modifications comprise acetylation, polyglutamylation, or phosphorylation [1]. How posttranslational modifications affect specific functions is largely unknown, although there is increasing evidence that modifications act as “traffic signs” for microtubule-dependent motor proteins [3]. Recently, it was shown that differences in the ratio between tyrosinated and detyrosinated alpha-tubulin in axons and dendrites confer directional cues for kinesin-1-dependent transport in axons [2], [4].

In lower eukaryotes only some alpha-tubulin modifications were identified and it appears that certain modifications arose at different times during evolution [1]. There is evidence that detyrosinated or otherwise modified MTs exist in the filamentous fungus *A. nidulans*. In two-dimensional gels four protein spots were identified as alpha-tubulin although only two genes are found in the *A. nidulans* genome. The same situation was found for beta-tubulin [5], [6]. Further evidence came recently from a study related to the kinesin 3 motor UncA [7]. Kinesin-3 motors contain the conserved motor domain, a FHA domain (forkhead homology-associated domain) involved in phosphorylation dependent protein-protein interactions, signaling pathways and the regulation of kinesin motors and a PH domain (Pleckstrin homology domain) at the carboxy terminus for cargo binding [8]. In *A. nidulans* and *Ustilago maydis* kinesin-3 is involved in vesicle trafficking, and deletion of the gene causes a reduction of the growth rate [7], [9]. Most surprisingly, UncA^rigor^ did not decorate all microtubules in a hyphal compartment of *A. nidulans* but only a subpopulation consisting of modified alpha-tubulin. An antibody against tyrosinated alpha-tubulin did not recognize the MT decorated by UncA^rigor^. This suggested that the modified MT might consist of detyrosinated alpha-tubulin [7]. However, direct biochemical evidence is not yet available. The exact cargo of UncA also remains to be defined. In *N. crassa* the motor is involved in mitochondrial distribution and in *U. maydis* in endosome trafficking [9], [10]. In *A. nidulans* there is evidence that UncA is involved in endosome movement and that endosomes are involved in polarized growth [11], [12]. We were meanwhile able to isolate vesicles associated to the UncA motor and are currently analyzing the protein content (own unpublished data).

Fascinating questions refer to the generation and maintenance of different MT populations, their different biological functions and the mechanism of motor-preference for one or the other MT population. Here, we present first evidence of how a kinesin-3 motor protein distinguishes between different MT populations in *A. nidulans*, and, surprisingly, how a short region in the tail of kinesin 3 is important for “traffic sign” recognition.

## Results

### The tail of UncA is necessary for microtubule specificity

**Figure 1:**
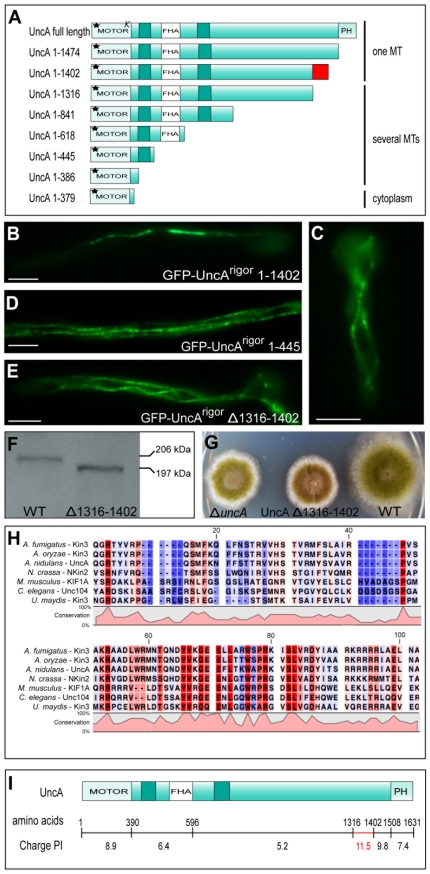
UncA-deletion analysis reveals that the tail of UncA is involved in specificity determination. (**A**) Scheme for the UncA-deletion analysis. The number of amino acids is given in front of the truncated proteins. Motor = motor domain containing the lysine-rich loop (*K*) and a rigor mutation in the P-loop (asterisk); FHA = forkhead associated domain; PH = pleckstrin homology domain. The red square indicates a 86 aa amino acid stretch. (**B–E**) Localization of different UncA truncated versions (as indicated) in the *ΔuncA* strain SNZ9. UncA proteins were labeled with GFP and expressed under the control of the *uncA* promoter. Scale bar, 5 µm. (**F**) Confirmation of expression levels by Western blot analysis of GFP-UncA^rigor^ (206 kDa)(SNZ14) and GFP-UncA^rigor^ *Δ*1316–1402 (194 kDa)(SCoS124). Western blot detection was done with anti-GFP antibodies (1∶4000) and anti-rabbit IgG peroxidase conjugated secondary antibodies (1∶4000). 285 ng crude protein extract was loaded. (**G**) Colonies of SNZ9, SCoS75 and wildtype (TN02A3). (**H**) Alignment of the 86 aa region of UncA orthologues from different fungi and higher eukaryotes. Done with CLC Sequence Viewer 6. See also Figure S1. (**I**) Calculation of isoelectric points for distinct regions of UncA.

The kinesin-3 motor UncA binds preferentially to modified microtubules (MTs) [7]. This was most obvious when a rigor mutation was introduced into the motor protein: a GFP-UncA^rigor^ fusion protein labeled mainly one bundle of MTs per compartment, whereas other kinesins, mutated and tagged in the same way, labeled several MTs. In order to identify the region that confers posttranslationally modified MT specificity, we performed a deletion analysis, starting from a full-length GFP-UncA^rigor^ version. Constructs shorter than 1316 amino acids bound to several MTs, whereas proteins longer than 1402 amino acids displayed specificity for modified MTs (**Fig. 1A–C**). From these results we concluded that the region between amino acid 1316 and 1402 is involved in modified MT selection. All eight different truncation constructs of UncA^rigor^ were tagged at the N-terminus with GFP, and expressed under the control of the *uncA* promoter in an *uncA*-deletion strain. The UncA tail region was compared to several kinesin-3 proteins, from fungi to humans, and showed several highly conserved residues (**Fig. S1**). In contrast, similar work in rat hippocampal neurons with kinesin 1 revealed a short region in the motor domain, designated the beta5-L8 Region, required for specificity [2]. Several residues within this region were essential for discrimination between tyrosinated and detyrosinated MTs; however, the corresponding region in UncA resembled regions in kinesins, which do not prefer detyrosinated MTs (data not shown).

Deletion analysis also revealed that the neck linker (amino acids 379–386), a mechanical element that has been shown to connect the motor domain with cargo and kinesin partner heads [13], was necessary for MT binding of UncA (**Fig. 1D**). Constructs only harboring the motor domain (379 amino acids), including the predicted MT-binding motif, were unable to bind MTs *in vivo* and localized to the cytoplasm; these results corroborate findings for *Caenorhabditis elegans* kinesin 3 (Unc104). This kinesin 3 undergoes concentration-dependent dimerization *in vitro* as a result of two short helical domains that are directly C-terminal to the neck linker [14]. The neck linker of mouse KIF5C (kinesin 1) can functionally and structurally replace the one of KIF1A [15]. Hence, the neck linker is an element that connects the motor domain to the cargo, or to another motor domain in the case of kinesin dimers, indicating that this element is essential for motor function.

Recently, Huckaba *et al.* showed that *Drosophila* kinesin 3, Khc-73, exists *in vitro* and *in vivo* in an equilibrium between monomer and dimer, is enriched at the ends of MTs, and is recruited to Rab5-containing vesicles [16]. In contrast, kinesin 3 from *Neurospora crassa,* NcKin3, was shown to be dimeric, but inactivates one of its motor heads to generate non-processive motility [17]. The data of Adio and Woehlke confirmed that the neck domain is required for dimerization and is essential for NcKin3 function: the absence of the neck altered the kinetic cycle fundamentally [18].

In order to further characterize the function of the 86 amino acids in the *A. nidulans* UncA tail and concurrently preserve as much of the UncA protein as possible, we deleted only this section of amino acids in the full-length protein expressed under the native promoter. This construct was transformed into an *uncA*-deletion strain in order to exclude homologous integration events into the *uncA* locus. We observed loss of specificity in this modified motor protein, confirming the role in specificity determination for modified MTs in the tail of UncA (**Fig. 1E**). Furthermore, we deleted the 86 amino acids in the original strain SNZ14 (*alcA*(p)::GFP::*uncA* ^rigor^), which has been previously used in localization studies [7]. This was achieved using a complementation strategy into the GPF-UncA^rigor^ strain, with homologous integration in the *uncA* locus (SNZ14) to exclude effects due to different protein expression levels. In full support of our hypothesis, UncA lacking the 86 amino acids decorated all MTs (data not shown). Similar expression levels of the GFP-UncA proteins in the two strains (SNZ14 and SCoS124) were confirmed by Western blot analysis (**Fig. 1F**). In order to test the functionality of the UncA protein lacking the 86 aa (SCoS75), colonies of this strain were compared to wildtype and to a *ΔuncA*-deletion strain. The fact that the colonies of SCoS75 showed the same compact colony phenotype, suggests that the 86 aa are required for function of UncA (**Fig. 1G**). An alignment of this stretch between different fungi and higher eukaryotes revealed many conserved amino acids in this region (**Fig. 1H**). In addition, this region is characterized by a positive charge at pH7 (**Fig. 1I**).

**Figure 2:**
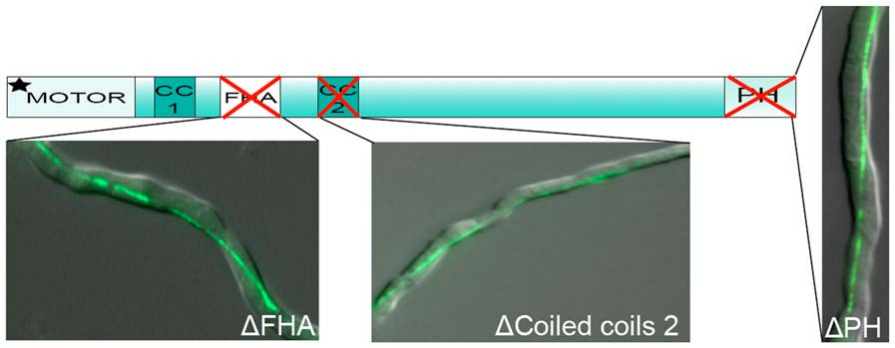
Analysis of UncA versions with deletions of the forkhead associated domain (FHA)(SCoS61), the pleckstrin homology domain (PH)(ScoS16), and the coiled coils (CC)(SCoS81) region. Hyphae are 3 µm in diameter.

In order to exclude a potential contribution of other domains in UncA to the determination of MT specificity, the FHA domain and the two coiled regions between the FHA and PH domains were deleted in the same way as the 86 amino acids: neither of these deletions affected the specificity (**Fig. 2**). The FHA (forkhead-associated) domain has been shown to be involved in phosphorylation-dependent protein-protein interactions, signaling pathways [19] and regulation of kinesin motors [20].

### The tail of UncA is sufficient for microtubule specificity

**Figure 3:**
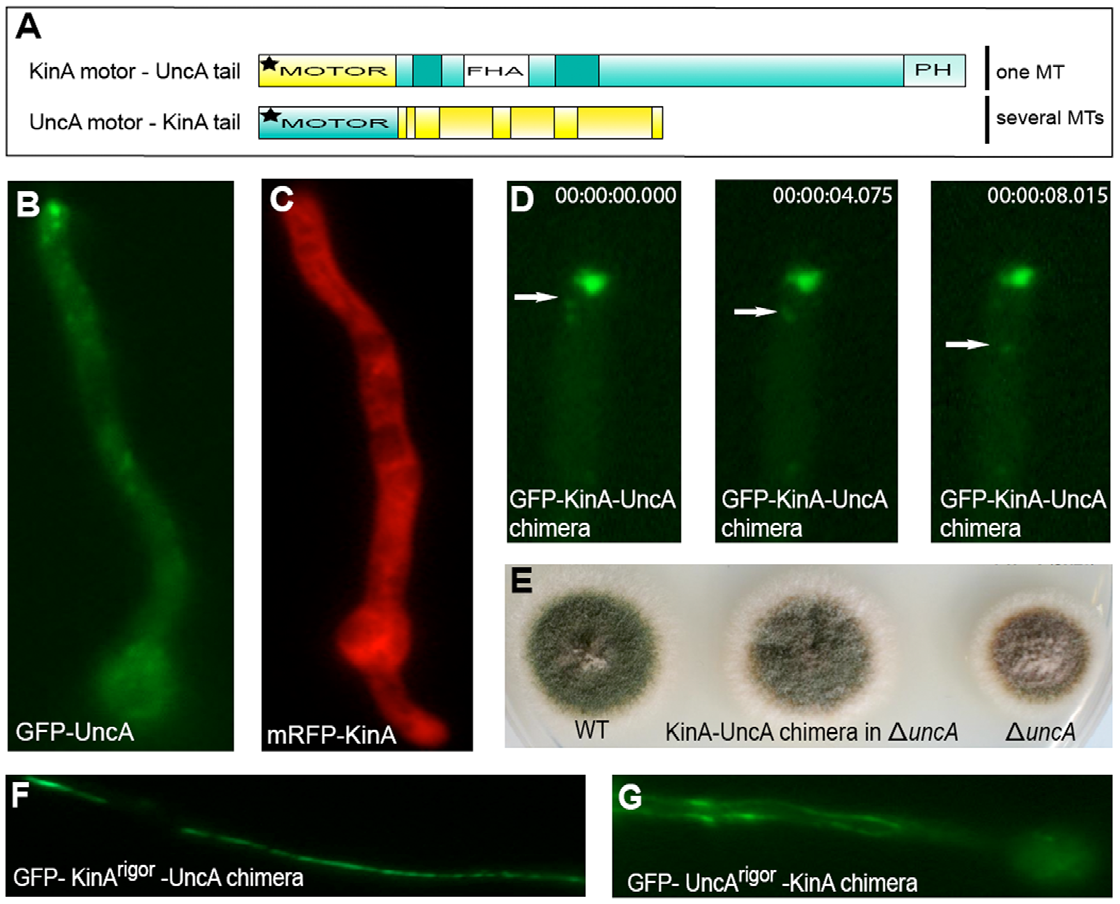
Chimeric kinesin proteins verify that the UncA tail is sufficient for microtubule specificity. (**A**) Scheme for the creation of chimera of kinesin 1 (yellow), KinA, and kinesin 3, UncA (green). (**B**) Localization of GFP-UncA (SNZ2) and (**C**) mRFP-KinA (SCS6-NZ). (**D**) Time lapse of the KinA–UncA chimeric protein (SCoS23). Arrows indicate a moving vesicle. Vesicles also accumulate at the tip of hyphae, similar to the UncA localization. (**E**) Growth comparison of WT, *ΔuncA* and the *ΔuncA* strain complemented with the KinA-UncA chimera (SCoS23). The fusion protein can restore the *ΔuncA* phenotype. (**F**) Localization pattern of KinA^rigor^-UncA chimera (SCoS24) labeled with GFP in the *ΔuncA* strain, under the control of the *uncA* promoter. The chimera shows the same specificity as UncA^rigor^. (**G**) In contrast UncA^rigor^-KinA chimera (SCoS44) in *ΔuncA*, labeled with GFP, under the control of the *uncA* promoter do not label MT subpopulations. Hyphae are 3 µm in diameter.

If the specificity of UncA is determined by the tail region of the motor, we asked ourselves whether the tail is capable of rendering conventional kinesin (KinA) specific for this MT subpopulation. To this end, we fused the KinA motor domain with the tail region of UncA under the control of the *uncA* promoter (**Fig. 3A**). Kinesin-1 and Kinesin-3 show different localization patterns in hyphae. Whereas the GFP-UncA fusion protein labels small bidirectional moving spots, which accumulate at the tip (**Fig. 3B**), KinA shows diffuse labeling of the whole cytoplasm (**Fig. 3C**). This has been observed before in *A. nidulans* and in *U. maydis*. Stable association of KinA with MTs was only observed after depletion of ATP or introduction of a rigor mutation [7], [21], [22]. Apparently the cytoplasmic concentration of KinA is quite high and association with MTs only temporarily. Futhermore, KinA transports – in addition to vesicles - also dynein towards the MT plus end [23]. These are likely only single molecules or dimers moving along MTs and thus cannot be visualized with normal epifluorescence microscopy of GFP-KinA. The generated chimeric protein KinA-UncA was N-terminally tagged with GFP and transformed into an *uncA-*deletion strain. Ectopic integration into the genome - to exclude integration into the *kinA* locus - was confirmed by Southern blot analysis (data not shown). The resultant strain showed vesicle movement and accumulation of the GFP signal at the tip of transformed hyphae (**Fig. 3D**), and the chimera was able to rescue the *uncA-*deletion phenotype, suggesting that the fusion protein is biologically active and transports probably UncA cargoes similar to the wildtype (**Fig. 3E**).

Subsequently to further analyze the specificity of this chimera, we created a strain with the rigor mutation at the ATP binding site of this chimeric motor; interestingly, this strain also displayed impressive specificity for the MT subpopulation (**Fig. 3F**), indicating that the tail of UncA is sufficient for generating MT specificity. As a further control, another chimeric motor protein was constructed by fusing the UncA^rigor^ motor domain to the tail of KinA. This fusion protein was also tagged with GFP at the amino terminus and expressed under the control of the *uncA* promoter to guarantee comparable expression levels. A loss of specificity was displayed, and several MTs were labeled (**Fig. 3G**). Likewise, a chimeric protein of the UncA^rigor^ motor domain and the tail of kinesin 7, KipA, did not show any preference for MT subpopulations (data not shown). Taken together, these findings suggest that the UncA tail is necessary for vesicle selection and MT specificity determination. The UncA motor domain appears only required for movement and can be replaced by the KinA motor domain.

### The tail of UncA is able to bind toalpha-tubulin

**Figure 4:**
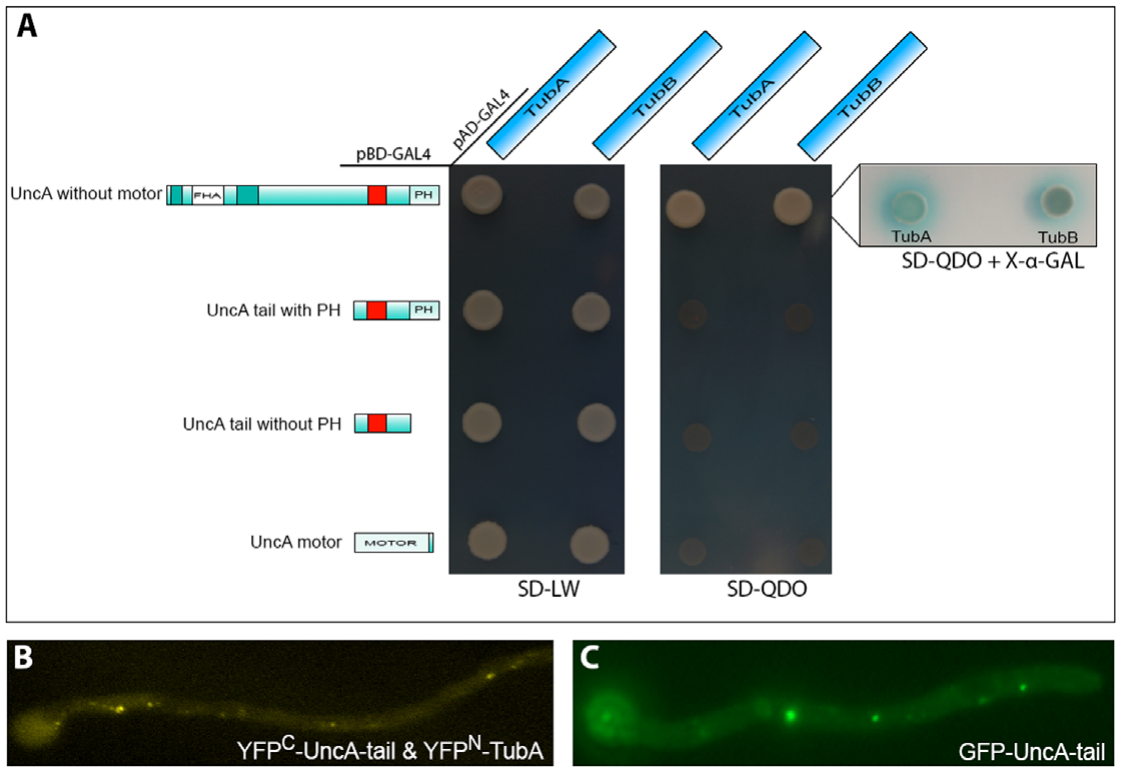
The tail of UncA is able to bind to alpha tubulin. (**A**) Yeast two-hybrid interaction tests with different truncations of UncA to map the interaction site between these proteins. Only the full-length tail region of UncA is able to interact with either of the two alpha tubulins. Transformants were assayed for growth on SD-LW to confirm integration of both constructs (left) and on SD-QDO for nutritional selection for positive interactions (right). The strength of the interaction is shown in the X-α-Gal assay. The red square indicates the 86 amino acids region. (**B**) Bimolecular fluorescence complementation assay with the YFP-C-terminal half fused to the UncA-tail and the YFP-N-terminal half fused to TubA in strain (SCoS126). (**C**) Subcellular localization of the GFP-UncA-tail in SCoS127. The tail of UncA localizes to vesicles, which moved in antero- and retrograde direction. Hyphae are 3 µm in diameter.

Because the tail of UncA is apparently able to interact with MTs or MT-associated proteins, we tested for interaction between alpha tubulin and UncA using the yeast two-hybrid assay. The bait of *uncA,* which spans from the end of the motor domain to the stop codon, was transformed into the *Saccharomyces cerevisiae* strain AH109. Transformed yeast was crossed to the compatible strain Y187 containing the corresponding full-length protein of either alpha tubulin, TubA or TubB. *A. nidulans* comprises two genes, *tubA* and *tubB*, which encode two isoforms of alpha tubulin. Both TubA and TubB showed an interaction with the tail of UncA (**Fig. 4A**). In principle, this interaction could be due to a bridging protein of *S. cerevisiae*. However, we confirmed the interaction using bimolecular fluorescence in *A. nidulans* (see below). In the resulting transformants spots of different sizes were labeled and moved anterograde and retrograde (**Fig. 4B**). This suggested that the UncA tail still binds to vesicles and is able to interact with cytoplasmic alpha-tubulin. The observed movement seems at a first glance curious, but the UncA tail still harbors the PH domain and is therefore probably still able to bind to its specific cargoes. These vesicles are not only transported by UncA but also by dynein [7]. To confirm the binding of the tail to vesicles, we analyzed the localization pattern of the UncA tail as GFP fusion protein (**Fig. 4C**). It revealed the same picture as in the bimolecular fluorescence complementation (BiFC) experiment.

In order to map the interaction site of UncA, we designed different truncations of UncA. Yeast two-hybrid assays between these proteins revealed that only the full-length tail region of UncA is able to interact with either of the two alpha tubulins. The interaction tests between the UncA motor domain and TubA or TubB were negative, indicating that either the motor domain is not able to bind monomeric alpha tubulin in the yeast two-hybrid assay, or the tail of UncA is necessary for proper motor domain folding, which enables binding. Interaction tests between the UncA motor domain and the tail of UncA were also negative, suggesting that intramolecular folding between motor and tail does not regulate activity or specificity. Such a regulatory mechanism has been shown before for kinesin-1, where the tail regulates the ATPase activity [24].

Taken together, the results suggest interaction between the tail of UncA and alpha-tubulin. Given that the interaction between the two proteins took place in the nucleus in the yeast-two hybrid assay and in the cytoplasm in *A. nidulans* in the BiFC analysis, suggests rather direct interaction than complex formation with other proteins.

## Discussion

Most eukaryotes contain several tubulin-encoding genes that generate alpha-tubulin with slightly different properties. In *A. nidulans,* for example, two genes encode for α and two for beta-tubulin [5], [6], [25]. Although deletion of one of the alpha-tubulin genes, *tubB*, was possible, this strain was subsequently unable to reproduce sexually; this defect was partially overcome via overexpression of *tubA*. These results demonstrate nicely overlapping but also distinct functions of certain tubulin isoforms; however, this picture becomes further complicated by the fact that a number of different posttranslational MT modifications exist. Already in 1975, Arce *et al.* reported the posttranslational incorporation of L-tyrosine into alpha-tubulin, indicating the presence of tyrosinated and detyrosinated MT forms [26]. alpha-tubulin generally ends with the tripeptide EEY, and the tyrosine residue is cyclically removed by carboxypeptidase, then re-added to the chain by tubulin-tyrosine ligase (TTL) [1]. Other possible modifications include acetylation, polyglutamylation, polyglycylation or phosphorylation [1]. In general, little is known about either modifying enzymes or the biological functions of these modifications, although the suppression of tubulin tyrosine ligase and subsequent accumulation of detyrosinated tubulin favors tumor growth in animal models and human cancers [27].

In neurons it was demonstrated that polarized trafficking of kinesin-1-driven vesicle movement is regulated through the balance between tyrosinated and detyrosinated MTs [2]. Since somatodendrites contain tyrosinated alpha-tubulin and the axon contains detyrosinated alpha-tubulin, inhibited binding of kinesin-1 to tyrosinated MTs restricted the transport function to the axons. In this case, the β5-L8 region in the motor domain was responsible for MT discrimination. In contrast, we show that in *A. nidulans* the 86 amino acid long region in the tail of kinesin-3 UncA was involved in the recognition process of modified, possibly detyrosinated MTs. Interestingly, UncA cargoes appear to be un-involved in MT recognition, since deletion of the PH domain did not alter its specificity (**Fig. 1**). In comparison, there is evidence that mammalian kinesin-1 cargo proteins may be involved in specificity determination in neuronal cells [28]. Unfortunately, no structural data are available yet for the tail of a kinesin-3 motor protein, because only the motor domain and its binding to MTs has been analyzed [29]. In comparison to our data, it was shown in mice that kinesin-3 uses polyglutamylation as a neural molecular traffic sign, although the structural mechanism of the kinesin remained enigmatic [30]. The picture becomes increasingly complex when considering the recent observation that the modification type may switch from detyrosination to acetylation upon polarization of epithelial cells [31]. It will be the challenge for future studies to unravel the exact mechanism(s) of motor proteins for MT discrimination between different MTs, and determine how posttranslational modifications contribute to navigational cues for different motor proteins.

## Methods

### Strains, Plasmids, and Culture Conditions

Supplemented minimal media (MM) for *A. nidulans* and standard strain construction procedures are described by Hill and Käfer (2001) [32]. A list of *A. nidulans* strains used in this study is given in **Table 1**. Standard laboratory *Escherichia coli* strains (XL1 blue, Top 10) were used. Plasmids are listed in **Table 2**. *S. cerevisiae* strains are listed in **Table 3**.

**Table 1:** *A. nidulans* strains used in this study.

Strain	Genotype	Source
TN02A3	*pyrG89*; *argB2*, *nkuA*::*argB*; *pyroA4*	Nayak *et al*. (2006)
SNZ2	TN02A3 transformed with pAS3 (*alcA*::sGFP::*uncA*), *pyroA4*	Zekert *et al*. (2009)
SNZ9	TN02A3 transformed with pNZ13 (*uncA* deletion), *pyrG89*	Zekert *et al*. (2009)
SNZ14	TN02A3 transformed with pNZ15 (GFP-UncA^rigor^), *pyroA4*	Zekert *et al*. (2009)
SCS6-NZ	TN02A3 transformed with pCS-NZ5 (mRFP-KinA^rigor^), *pyrG89*	Zekert *et al*. (2009)
SCoS15	SNZ9 transformed with pCoS35 (*uncA*(p)*::sGFP::uncA* ^rigor^ *1–1316*); *veA1*	this study
SCoS16	SNZ9 transformed with pCoS38 (*uncA*(p)::sGFP::*uncA* ^rigor^1–1402); *veA1*	this study
SCoS21	SNZ9 transformed with pCoS25 (*uncA*(p)::sGFP::*uncA* ^rigor^1–445); *veA1*	this study
SCoS23	SNZ9 transformed with pCoS44 (*uncA*(p)::sGFP::*kinA*1–348::*uncA*373–1630); *veA1*	this study
SCoS24	SNZ9 transformed with pCoS46 *(uncA*(p)::sGFP::*kinA* ^rigor^1–348::*uncA*373–1630); *veA1*	this study
SCoS44	SNZ9 transformed with pCoS61 *(uncA*(p)::sGFP::*uncA* ^rigor^_r_1–379::*kinA*343–927); *veA1*	this study
SCoS57	SNZ14 transformed with pCoS73 (Q1314stop complementation); *pyrG89*; *veA1*	this study
SCoS58	SNZ14 transformed with pCoS72 (A1402stop complementation); *pyrG89*; *veA1*	this study
SCoS61	SNZ9 transformed with pCoS80 (*uncA*(p)::sGFP::*uncA* ^rigor^without FHA); *veA1*	this study
SCoS62	SNZ9 transformed with pCoS81 (*uncA*(p)::sGFP::*uncA* ^rigor^without CC2+CC3); *veA1*	this study
SCoS75	SNZ9 transformed with pCoS75 (*uncA*(p)::sGFP::*uncA* without 86 aa)); *pyrG89*; *veA1*	this study
SCoS124	SNZ14 transformed with pCoS135 (complementation without 86 aa); *pyrG89; veA1*	this study
SCoS126	TN02A3 transformed with pCoS155 and pCoS151 (*alcA*(p)::YFP^C^::unc*A* ^full length tail^, *alcA*(p)::YFP^N^::TubA; *veA1*	this study
SCoS127	TN02A3 transformed with pCoS156 (*alcA*(p)::GFP::unc*A* ^full length tail^); *pyroA4*; *veA1*	this study

**Table 2:** Plasmids used in this study.

Plasmid	Construction	Source
pCR2.1-TOPO	Cloning vector	Invitrogen
pCMB17apx	*alcA*(p)::GFP, for N-terminal fusion of GFP to proteins of interest; contains *N. crassa pyr4*	Efimov *et al.* (2006)
pGBKT7	Yeast Two-Hybrid bait vector, Gal4-BD	Clontech
pGADT7	Yeast Two-Hybrid prey vector, Gal4-AD	Clontech
pNZ-SI49	1.5-kb *uncA*(p) fragment in pAS3 with *Kpn*I-*EcoR*I sites	Zekert *et al.* (2009)
pNZ-SI71	*alcA*(p)::mRFP::*uncA* ^full length^ with *Asc*I-*Pac*I sites in pCMB17apx	Zekert *et al.* (2009)
pNZ15	pAS3 mutagenesis to introduce the G116E mutation in the p-loop of UncA, (UncA^rigor^)	Zekert *et al.* (2009)
pCS4-NZ	pCS2-NZ mutagenesis to introduce the G97E mutation in the p-loop of KinA, (KinA^rigor^)	Zekert *et al.* (2009)
pCoS25	*uncA*(p)::sGFP::*uncA* ^rigor^1–445 with *Asc*I-*Pac*I sites in pCMB17apx	this study
pCoS35	*uncA*(p)::sGFP::*uncA* ^rigor^1–1316 with *Asc*I-*Pac*I sites in pCMB17apx	this study
pCoS38	*uncA*(p)::sGFP::*uncA* ^rigor^1–1402 with *Asc*I-*Pac*I sites in pCMB17apx	this study
pCoS44	*uncA*(p)::sGFP:*:kinA*1–348::*uncA*373–1630 with *EcoR*I-*Pac*I sites in pCMB17apx	this study
pCoS46	*uncA*(p)::sGFP::*kinA* ^rigor^1–348::uncA373–1630 with *EcoR*I-*Pac*I in pCMB17apx	this study
pCoS61	*uncA*(p)::sGFP::*uncA* ^rigor^1–445::*kinA*343–928 with *EcoR*I-*Pac*I sites in pCMB17apx	this study
pCoS72	SNZ14 complementation construct A1402stop mutation (AscI-*uncA*-tail-PacI-*pyro*-NotI-*uncA*-RB-AscI) in Topo2.1	this study
pCoS73	SNZ14 complementation construct A1316stop mutation (AscI-*uncA*-tail-PacI-*pyro*-NotI-*uncA*-RB-AscI) in Topo2.1	this study
pCoS75	*uncA*(p)::sGFP::*uncA* ^rigor^ without 86 aa (1316–1402) with *Asc*I-*Pac*I sites in pCMB17apx	this study
pCoS80	*uncA*(p)::sGFP::*uncA* ^rigor^ without FHA (495–596) with *Asc*I-*Pac*I sites in pCMB17apx	this study
pCoS81	*uncA*(p)::sGFP::*uncA* ^rigor^ without CC2 (679–823) with *Asc*I-*Pac*I sites in pCMB17apx	this study
pCoS82	*tubA* with *Xba*I-*Cla*I sites in pGADT7	this study
pCoS84	*tubB* with *Xba*I-*Cla*I sites in pGADT7	this study
pCoS85	*uncA* tail without PH (951–1497) with *EcoR*I-*Sma*I sites in pGBKT7	this study
pCoS86	*uncA* tail with PH (951–1631) with *EcoR*I-*Sma*I sites in pGBKT7	this study
pCoS87	*uncA*(p)::sGFP::*uncA* ^rigor^1–390 with *EcoR*I-*Sma*I sites in pGBKT7	this study
pCoS135	SNZ14 complementation construct without 86 aa (without 1316–1402) (*Asc*I-*uncA*-tail-*Pac*I-pyro-*Not*I-*uncA*-RB-*Asc*I) in Topo2.1	this study
pCoS151	*alcA*(p)::YFP^N^::*tubA^3,1 kb^* with *Asc*I-*Pac*I sites in pCMB17apx	this study
pCoS155	*alcA*(p)::YFP^C^::*uncA^full-length^* ^ tail^ with *Asc*I-*Pac*I sites in pCMB17apx, *pyro* instead of *pyr4*	this study
pCoS156	*alcA*(p)::sGFP::*uncA^full-length^* ^ tail^ with *Asc*I-*Pac*I sites in pCMB17apx	this study

**Table 3:** *S. cerevisiae* strains used in this study.

Strain	Genotype	Source
AH109	*MATá, trp1901,leu2–3,112, ura3–52,his3–200,gal4Ä, gal80Ä, LYS2:: GAL1_UAS_-GAL1_TATA_-His3, GAL2_UAS_-GAL2_TATA_-Ade2, URA3::MEL1_UAS_-MEL1_TATA_-lacZ, MEL1*	Clontech
Y187	*MATá, ura3–52, his3–200, ade2–101, trp1–901, leu2–3, 112, gal4Ä, met–, gal80Ä, MEL1, URA3::GAL1_UAS_ -GAL1_TATA_-lacZ*	Clontech
yCoS1	Y187 transformed with pCoS82 (*tubA* in pGADT7)	this study
yCoS3	Y187 transformed with pCoS84 (*tubB* in pGADT7)	this study
yCoS4	AH109 transformed with pCoS85 (*uncA* tail without PH (951–1497) in pGBKT7)	this study
yCoS5	AH109 transformed with pCoS86 (*uncA* tail with PH (951–1631) in pGBKT7)	this study
yCoS6	AH109 transformed with pCoS87 (uncA^rigor^motor in pGBKT7)	this study
yCoS8	AH109 transformed with pCoS89 (*uncA* without motor (352–1631) in pGBKT7)	this study

### Molecular Techniques

Standard DNA transformation procedures were used for *A. nidulans* [33] and *E. coli* [34]. For polymerase chain reaction (PCR) experiments, standard protocols were applied. DNA sequencing was performed commercially (Eurofins MWG Operon Ebersberg, Germany). DNA analyses (Southern hybridizations) were performed as described [34].

Domain deletion of UncA was achieved using primers with phosphorylated 5′-ends to amplify the entire vector pCoS21, with the exception of the deleted region. The primers used for the FHA mutant were del_FHA_fwd (P-5′-CCC CAG GAA GCA AGG GCT GAA C-3) and del_FHA_rev (P-5′-CTT TTT CGG TGT ACT CAA ACC GAT AAA TCC-3); deletion of 80 aa stretch were done using del80_mut_fwd (P-5′-GTG CAG GGG CGG CGT ATT GCA GAG CTT AAC G-3) and (P-5′-CGT TAA GCT CTG CAA TAC GCC GCC CCT GCA C-3); the coiled coil deletion used del_CC2+3_fwd (P-5′-CAC CCA GTG CCA AGA ATC TAC GAG AAT G-3) and del_CC2+3_rev (P-G AGC AAG CCT ATC CGG ATC CAT C -3). All plasmids were transformed into the Δ*uncA* strain SNZ9.

### Tagging Proteins with Green Fluorescent Protein (GFP)

In order to create N-terminal GFP fusion constructs of UncA truncations, 1.2 to 4.4-kb long N-terminal fragments of *uncA* (starting from ATG) were amplified from pNZ-SI71 with the primers uncA_*Asc*_fwd1 (5-GGGCGCGCCCGGCATGGCGCCAGGAGGTGGTG-3) and several *Pac*I reverse primers. The *Asc*I-*Pac*I-digested PCR fragment was cloned into the corresponding sites of pCMB17apx, yielding truncated versions of UncA. To produce UncA N-terminal tagged with GFP under the native promoter, a 1.5-kb fragment of the putative *uncA* promoter was amplified from genomic DNA with the primers UncA nat(P)_*EcoR*I_fwd (5-GGA ATT CTC ATC ACC TAC TGG AGG CGC GC-3) and UncA_nat(p)_*Kpn*I rev (5-CGG TAC CTT TGG CCT ATA GCC CAT ACA CC-3), digested with *EcoR*I and *Kpn*I, and the two fragments were ligated with *EcoR*I-*Kpn*I–digested pAS3, yielding pNZ- SI49 (*alcA* promoter replaced with the *uncA* promoter in pAS3). All plasmids were transformed into Δ*uncA* strain SNZ9. Integration events were confirmed by PCR and Southern blot (results not shown).

### Creation of *uncA^rigor^* and *kinA^rigor^* Mutant Alleles

We changed the UncA glycine residue 116 to glutamate by site-directed mutagenesis using the oligonucleotides UncA_P-Loop_Gly_fwd (5-GGT CAG ACC GGT TCG GAG AAG TCT TAC TCG-3) and UncA_P-Loop_Gly_rev (5-CGAGTAA- GACTTCTCCGAACCGGTCTGACC-3), with plasmid pNZ-SI71 as a template, and the QuickChange XL site-directed mutagenesis kit (Stratagene, Heidelberg, Germany); this yielded plasmids for the truncation series. These primers were also used to generate the rigor mutations in chimeric motor proteins. The plasmids were sequenced to confirm the mutagenesis event and then transformed into the *uncA* deletion strain SNZ9. PCR and Southern blot analysis confirmed that the constructs were integrated ectopically. The same procedure was applied for KinA, using the primers KinA_Rigor_P-Loop_for (5-C GGT CAA ACC GGT GCA GAG AAG TCG TAT AC-3) and KinA_Rigor_P-Loop_rev (5-GT ATA CGA CTT CTC TGC ACC GGT TTG ACC G-3) to change glycine residue 97 to glutamate using pCS4-NZ as template.

### Protein extracts and Western blotting

For preparing protein extracts, *A. nidulans* strains were incubated in liquid MM for 24 h at 37°C. To induce the *alcA* promoter this medium was supplemented with 0.2% glucose and 2% threonine. The mycelium was harvested by Filtration through Miracloth (Calbiochem, Heidelberg, Germany), dried between some paper towels and immediately ground in liquid nitrogen. Afterwards the mycelial powder was resuspended in protein extraction buffer (20 mM Tris– HCl, pH 8, 0.05% Triton-X-100, 150 mM NaCl) containing protease inhibitors (1 mM PMSF). Cell debris was pelleted by centrifugation. After denaturation of samples protein extracts were loaded on a 7.5% sodium dodecyl sulfate polyacrylamide gel. For western blotting anti-GFP antibodies (anti-GFP N terminus, derived from a rabbit, product G1544; Sigma-Aldrich, Munich, Germany) were used and detected with anti-rabbit IgG peroxidase conjugate secondary antibody (product A0545; dilution, 1∶4,000; Sigma- Aldrich, Munich, Germany). 285 ng of protein extract was loaded. For blotting nitrocellulose membranes from Schleicher and Schuell (Dassel, Germany) were used.

### Light and Fluorescence Microscopy

For live-cell imaging of germlings and young hyphae, cells were grown on coverslips in 0.5 ml of MM 2% glycerol (de-repression of the *alcA* promoter, moderate induction). Cells were incubated at room temperature for 1 d. Images were captured at room temperature (200-ms exposure time) using an Axio Imager Z1 microscope (Carl Zeiss, Jena, Germany). Images were collected and analyzed with the AxioVision system (Carl Zeiss).

### Yeast Two-Hybrid Analysis

The yeast two-hybrid analysis was performed using the Matchmaker Library Construction & Screening system (BD Clontech). For strain generation, an *uncA* cDNA fragment corresponding to the C-terminal half of UncA (952–1630 amino acids) with UncA3kb_fd_*EcoR*I (5′-CGA ATTCATGAGGCAACTGCACCAGTAC-3′) and UncAfull_*Sma*I_rev (5′-GTTCCCGGGTCA TCTCCCGGACCTGTTG-3′) was amplified and cloned in the pGBKT7 vector, which contains the GAL4 DNA-BD and *TRP1* marker (BD Clontech). cDNA of *tubA* and *tubB* from *Aspergillus* strain TN02A3 were amplified and cloned in the pGADT7-Rec vector, which contains the GAL4 DNA-AD and the *LEU2* marker (BD Clontech). pGBK7-associated plasmids were transformed in yeast AH109 (mating type *MAT*a), whereas pGADT7-associated plasmids were transformed in yeast Y187 (mating type *MATalpha*). The system utilizes two reporter genes (*HIS3* and *LacZ*) under the control of the GAL4-responsive UAS. Beta-galactosidase activity was analyzed using the colony-lift filter assay with X-Gal (5-bromo-4-chloro-3-indolyl-β-D-galactopyranoside (Karl Roth)) as substrate. Interaction was quantified in a X-Gal assay, which detects the activation of the yeast *MEL1* gene, a GAL4-regulated gene used in two-hybrid analyses. *MEL1* encodes the secreted enzyme alpha-galactosidase, which hydrolyzes colorless X-Gal into a blue end product.

## Supporting Information

Figure S1 **Alignment of seven kinesin 3 proteins.** The alignment was done using CLC Sequence Viewer 6 with standard settings.
(PDF)
