# Supplementary figures and images for: The Aspergillus nidulans Kinesin-3 Tail Is Necessary and Sufficient to Recognize Modified Microtubules
Source: PLoS One. 2012 Feb 20;7(2):e30976. doi: 10.1371/journal.pone.0030976 (PMC3282709; doi:10.1371/journal.pone.0030976)

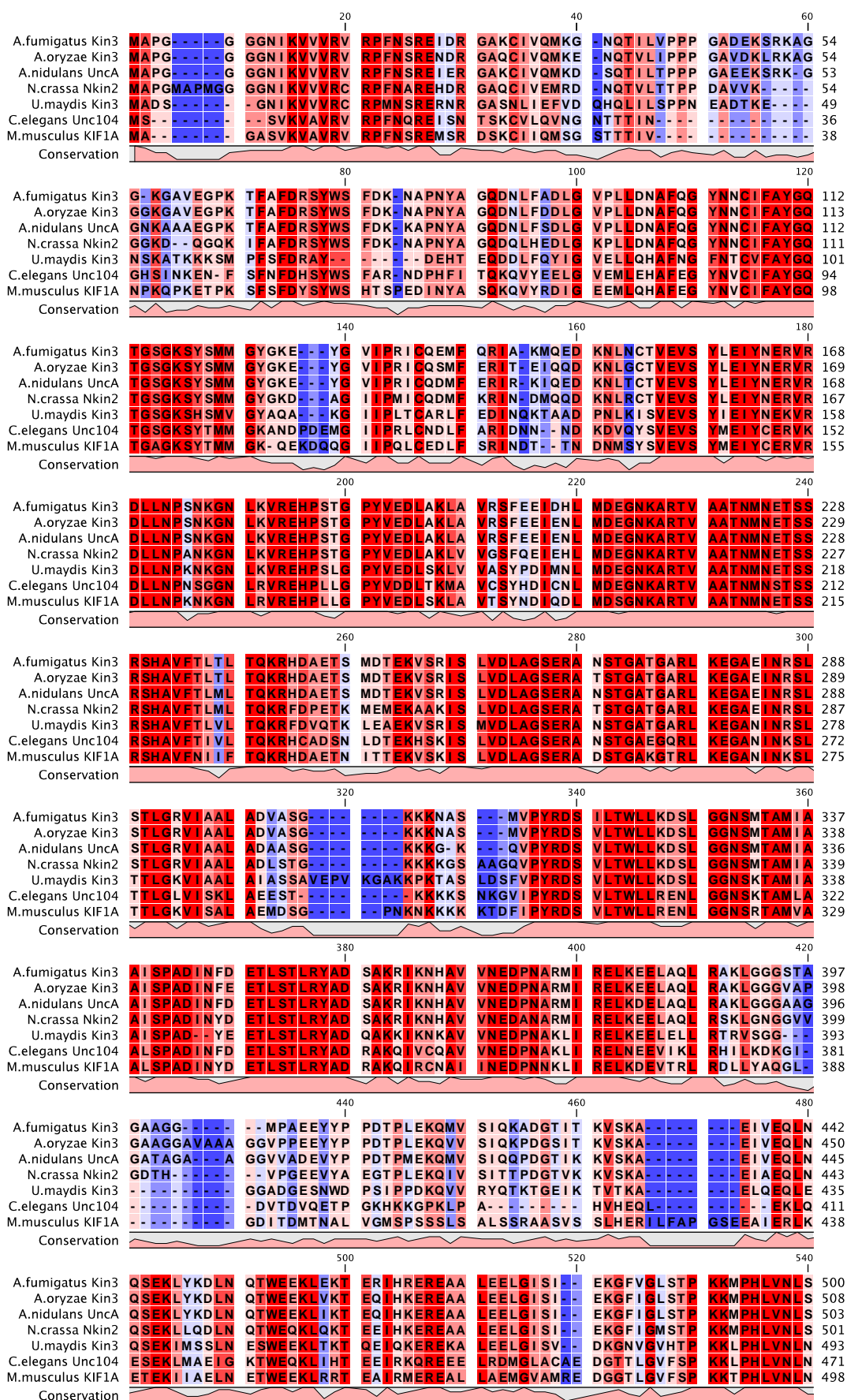

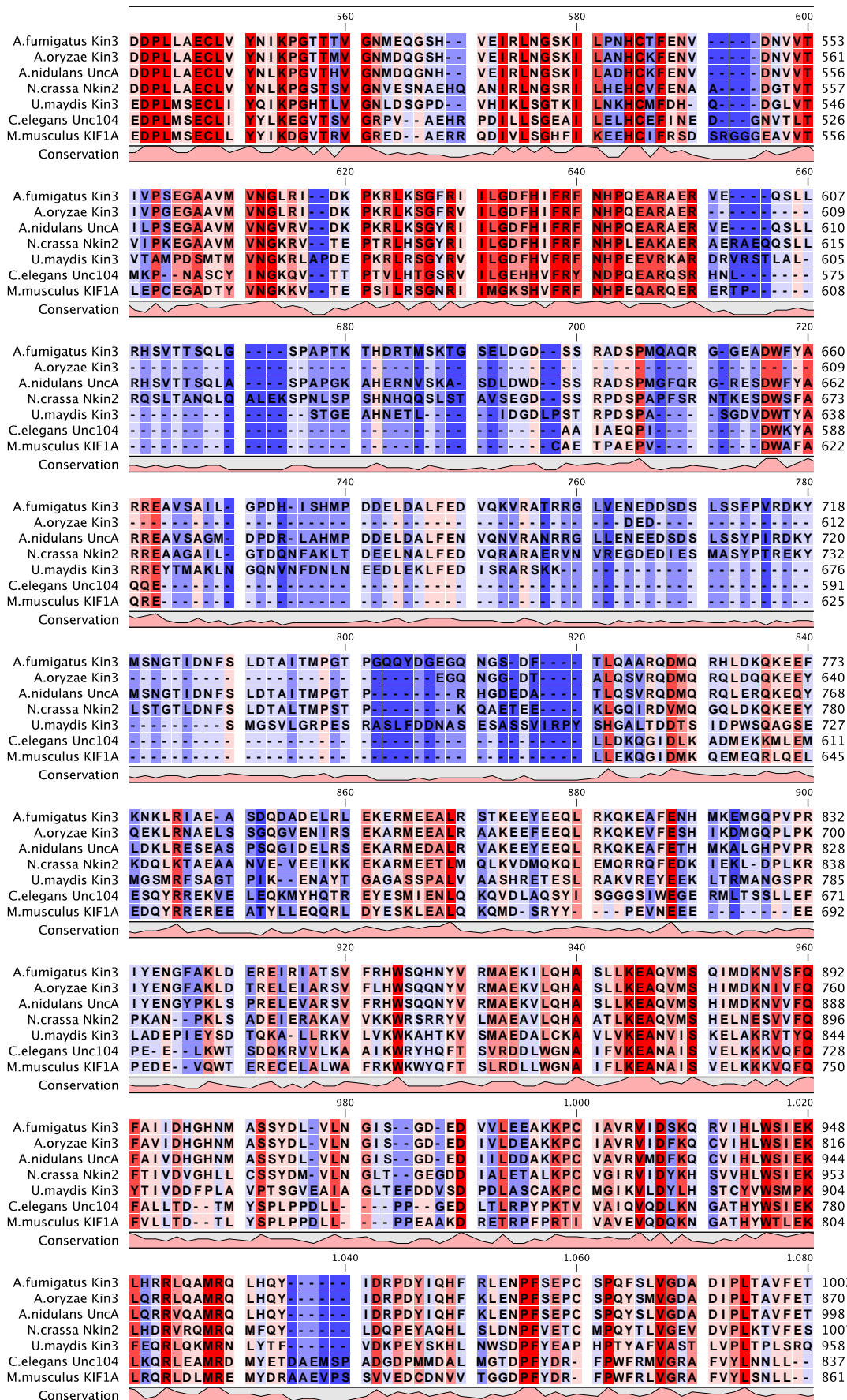



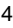

Supplement: Figure S1 — Alignment of seven kinesin 3 proteins. The alignment was done using CLC Sequence Viewer 6 with standard settings. (PDF) [file pone.0030976.s001.pdf]
